# Healthcare-associated infections after elective surgery for gastrointestinal cancer in a Romanian tertiary center: incidence, microbiological profile, and the role of neoadjuvant radiotherapy as an independent risk factor

**DOI:** 10.3389/fpubh.2026.1894824

**Published:** 2026-08-05

**Authors:** Ioana-Madalina Fieraru, Raluca-Maria Hrisca, Ion Stefan, Adriana Hristea

**Affiliations:** 1 “Carol Davila” University of Medicine and Pharmacy, Bucharest, Romania; 2 Department of Infectious Diseases, Central Military Emergency University Hospital “Dr. Carol Davila”, Bucharest, Romania; 3 Department for Prevention and Surveillance of Healthcare-Associated Infections, Central Military Emergency University Hospital “Dr. Carol Davila”, Bucharest, Romania; 4 Faculty of Medicine, Titu Maiorescu University, Bucharest, Romania; 5 National Institute for Infectious Diseases “Prof. Dr. Matei Balș”, Bucharest, Romania

**Keywords:** antimicrobial resistance, colorectal cancer, gastric cancer, healthcare-associated infection, neoadjuvant radiotherapy, perioperative antibiotic prophylaxis, surgical site infection, surveillance

## Abstract

**Background:**

Healthcare-associated infections following gastrointestinal cancer surgery are typically reported in terms of surgical site infection alone, with limited data on the broader infectious spectrum, comparative microbiology between upper and lower gastrointestinal surgery, and antimicrobial resistance in Eastern European centers. We aimed to describe the incidence, types, risk factors, and microbiological profile of all healthcare-associated infections occurring within 30 days of surgery in a Romanian tertiary referral center for oncological surgery.

**Methods:**

In a prospective single-center observational study, 211 consecutive adult patients undergoing elective surgery for gastric or colorectal cancer between May 2023 and July 2024 were followed for 30 days postoperatively. Healthcare-associated infections were defined according to Centers for Disease Control and Prevention/National Healthcare Safety Network surveillance criteria, and multidrug- and extensively drug-resistant isolates were classified using consensus international definitions. Risk factors were assessed by univariate analysis and multivariable logistic regression with three pre-specified predictors: neoadjuvant radiotherapy, surgery duration, and male sex.

**Results:**

Thirty-three patients (15.6%) developed at least one healthcare-associated infection, accounting for 39 episodes. The infection profile differed by surgical site: surgical site infection was the predominant infection type following lower gastrointestinal surgery (~46% of episodes), whereas device-associated and respiratory infections were relatively more common following upper gastrointestinal surgery (~38% of episodes together). Neoadjuvant radiotherapy-containing treatment was an independent risk factor in colorectal patients (adjusted odds ratio [OR] 3.25, 95% confidence interval [CI] 1.15–9.19, *p* = 0.026). Of 27 culture-positive isolates, 44.4% were classified as multidrug- or extensively drug-resistant, including a dual-carbapenemase-producing *Klebsiella pneumoniae*, a metallo-*β*-lactamase-producing *Pseudomonas aeruginosa*, and a single *Candida auris* isolate. Microbiological documentation was missing for 36.4% of infected patients, and infection-attributable 30-day mortality was 1.9%.

**Conclusion:**

Postoperative healthcare-associated infection in gastrointestinal cancer surgery extends substantially beyond surgical site infection and carries a high antimicrobial resistance burden in this Romanian tertiary-center cohort. Neoadjuvant radiotherapy warrants heightened postoperative surveillance, though this independent association requires confirmation in larger multicenter studies. The substantial proportion of culture-negative or unsampled infections highlights a concrete quality-improvement target, while the documented resistance profile indicates that institution-specific surveillance data may complement broader national or European epidemiological information when developing local stewardship strategies.

## 1 Introduction

Healthcare-associated infections (HAI) remain a significant burden in acute care settings: in the most recent European point-prevalence survey (2022–2023), an estimated 4.3 million patients in EU/EEA acute care hospitals acquired at least one HAI per year, with surgical site infections (SSI) accounting for only 16.1% of all HAI (1, 2). Beyond their frequency, they prolong hospitalization, increase morbidity and mortality, and add substantially to healthcare costs, while also acting as an important reservoir for the spread of antimicrobial resistance. Cancer patients are especially at risk given their immunocompromised status, numerous and prolonged hospitalizations, and complex treatments (3). Gastric and colorectal cancers rank among the five most common malignancies worldwide (4), often require surgical treatment, and are associated with a high incidence of SSIs (5, 6). According to GLOBOCAN 2022 estimates, gastrointestinal (GI) malignancies constitute a substantial cancer burden in Romania. Colorectal cancer was the most commonly diagnosed malignancy, accounting for 13,541 new cases, nearly 13% of all cancers, and was the second leading cause of cancer mortality, responsible for 7,381 deaths in 2022. Gastric cancer remains a significant health burden in Romania, with 3,823 new cases and 3,186 deaths reported in 2022. Although it ranked sixth in incidence, its relatively high mortality highlights the importance of early detection and effective treatment (7). The burden of both colorectal and gastric cancers underscores the need for prevention strategies, timely diagnosis, and improved access to cancer care services in Romania. While colorectal cancer has a higher incidence and prevalence, gastric cancer continues to have a considerable impact on cancer mortality, reflecting differences in disease detection and outcomes. Surgical resection remains the mainstay of curative treatment for both tumor types, yet it exposes an already vulnerable, frequently immunosuppressed population to major operative trauma and to contamination of the operative field by endogenous GI flora—conditions that together predispose to a range of postoperative infections.

The majority of published studies focus on SSI, which represents only a subset of postoperative infectious complications (8). Other HAI types—bloodstream infections, ventilator-associated pneumonia (VAP), catheter-related infections, and *Clostridioides difficile* infection (CDI)—are frequently encountered in this population but remain less extensively investigated than SSIs in surgical oncology populations. Because major gastrointestinal cancer operations frequently entail prolonged intensive-care stays, mechanical ventilation, and indwelling central venous and urinary catheters, in addition to disruption of the gut mucosal barrier, the true postoperative infectious burden extends well beyond the surgical wound, and a focus restricted to SSI risks substantially underestimating it. Consistent with this, in the international VISION cohort of adults undergoing noncardiac surgery, postoperative infection occurred in 9.8% of patients, with surgical site infection accounting for fewer than half (39.8%) of infectious episodes (8). Furthermore, comparative data on the microbiological profile of postoperative infections between upper and lower GI cancer surgery are scarce, particularly from Eastern European centers where the burden of antimicrobial resistance is substantial (9).

Comprehensive, prospectively collected data capturing the full spectrum of HAI together with their microbiological and resistance profiles are therefore needed to guide perioperative infection prevention and empirical therapy in this setting, particularly where antimicrobial resistance is high (10, 11) and prospective surveillance remains limited. The primary objective of this study was to describe the incidence, types, risk factors, and microbiological profile of all HAI occurring within 30 days of surgery in a prospective cohort of 211 patients undergoing gastric or colorectal cancer surgery in a Romanian tertiary referral center. A secondary, exploratory objective was to compare the infection landscape between upper and lower GI tract surgery.

## 2 Materials and methods

### 2.1 Study design and population

This was a prospective single-center observational study conducted in the General Surgery Departments of Central Military Emergency University Hospital “Dr. Carol Davila,” a tertiary referral center for oncological surgery in Bucharest, Romania. All adult patients undergoing elective surgery for histologically confirmed GI cancer between May 2023 and July 2024 were consecutively included and followed for 30 days after surgery (upper GI: gastric and small intestine surgery; lower GI: colorectal surgery). Gastric and small intestine cancers were grouped as upper GI given their shared proximal GI location, in contrast to colorectal (lower GI) surgery. Patients operated on for GI cancer in emergency settings were not eligible for inclusion due to their distinct perioperative risk profile and infection risk.

The study protocol was approved by the institutional ethics committee of Central Military Emergency University Hospital “Dr. Carol Davila” (approval number: 506/01.03.2022), as part of the institutional project “Prevention and Management Tools for Reducing Antibiotic Resistance.” Given the observational, non-interventional design and the use of routinely collected clinical and microbiological data, the requirement for written informed consent was waived by the ethics committee in accordance with national legislation and institutional requirements. Patient identifiers were removed prior to analysis. The study is reported in accordance with the STROBE guidelines for observational studies (12).

### 2.2 Data collection and outcome definitions

Demographic variables [age, sex, body mass index (BMI)], comorbidities [diabetes, chronic kidney disease (CKD), smoking status], surgical history (prior GI surgery, hospitalization in the previous 12 months), neoadjuvant treatment (radiotherapy, chemotherapy, or combined), American Society of Anesthesiologists (ASA) physical status class, surgical variables (duration and type of surgery, surgical ward), perioperative antibiotic prophylaxis (PAP) regimen and duration, and hospitalization outcomes (length of stay) were collected prospectively using a standardized electronic database. Patients were assessed at scheduled follow-up visits at 7, 14–21, and 30 days postoperatively, conducted either in person or by telephone. No *a priori* sample-size calculation was performed; the final sample of 211 patients represents a convenience sample defined by the recruitment window.

The primary outcomes were the occurrence of any HAI within 30 days of surgery and infection-attributable 30-day mortality. Infection-attributable death was adjudicated by the first author and defined *a priori* as death in a patient meeting the Sepsis-3 consensus criteria for sepsis or septic shock (13) due to an infection occurring within 30 days of surgery. HAI was defined according to Centers for Disease Control and Prevention/National Healthcare Safety Network (CDC/NHSN) surveillance criteria (14) as an infection with date of event on or after the third calendar day of admission. The following infection types were included: SSI, defined as infection at the operative site within 30 days of surgery per CDC/NHSN criteria (15); CDI, diagnosed by positive glutamate dehydrogenase (GDH) antigen and toxin assay; hospital-acquired pneumonia and VAP, defined per Infectious Diseases Society of America/American Thoracic Society (IDSA/ATS) criteria (16); primary bloodstream infection, requiring at least one positive blood culture with a recognized pathogen; central venous catheter (CVC) infection; intra-abdominal infections including peritonitis, cholecystitis, and postoperative septic complications requiring surgical re-exploration in the absence of SSI meeting CDC/NHSN criteria; and enterocolitis with negative *C. difficile* testing.

An infectious episode was defined as one distinct infection type per patient. The same infection type at multiple follow-up visits was counted as one persistent episode. Different infection types in the same patient were counted as separate episodes. This episode-counting rule was an institutional convention adopted for this study rather than a formally validated standard. It is consistent with the type-specific event definitions of CDC/NHSN surveillance (14); the decision to count repeated documentation of the same infection type as a single persistent episode, and different infection types as separate episodes, was an institutional operational choice applied uniformly to all patients.

For patients who developed HAI, microbiological data were recorded at each follow-up timepoint, including sample type, organism identification, and antimicrobial susceptibility profile. The lack of microbiological samples was also documented. The prospective database recorded whether microbiological documentation was present but did not capture sampling decisions as a discrete field; distinguishing unsent samples from negative cultures would have required a retrospective audit of nursing and laboratory source records across multiple wards and the post-discharge follow-up period, which could not be performed reliably for the full cohort and was therefore not undertaken.

Secondary outcomes included (i) the proportion of infected patients with microbiological documentation (categorized as culture-positive, diagnostic test only, or no sample obtained); (ii) the proportion of multidrug-resistant (MDR) or extensively drug-resistant (XDR) isolates among culture-positive infections as defined by Magiorakos et al. (17); (iii) pathogen distribution by Gram classification and species, stratified by surgical site; and (iv) the association between pathogen characteristics (including MDR/XDR status and Gram classification) and 30-day mortality. Intrinsic resistance for *Enterobacterales, Pseudomonas* spp. and *Acinetobacter* spp. was assessed according to European Committee on Antimicrobial Susceptibility Testing (EUCAST) expert rules (v3.2) (18).

### 2.3 Statistical analysis

Continuous variables were described as medians with interquartile ranges (IQR) and compared between groups using the Wilcoxon rank-sum test, chosen over the *t*-test due to non-normal distributions and small subgroup sizes. Categorical variables were described as frequencies and percentages. For 2 × 2 tables, Fisher’s exact test was used, as expected cell counts below 5 were frequent given the limited number of infection events. For variables with more than two categories, a permutation-based exact test (10,000 iterations) was used when expected cell counts fell below 5. Permutation-based exact tests were used for these variables because the asymptotic chi-square approximation is unreliable when expected cell counts are small. Because the univariate comparisons in Tables 1, 2 were exploratory and not adjusted for multiple testing, the corresponding *p*-values should be interpreted as descriptive and hypothesis-generating, with formal inference based on the pre-specified multivariable model.

**Table 1:** Patient characteristics by healthcare-associated infection (HAI) status following lower gastrointestinal surgery for colorectal cancer.

Variable	Total (*n* = 161)	No HAI (*n* = 138)	HAI (*n* = 23)	*p-*value
Age (years)	69 (63–75)	69 (62–75)	70 (66–74)	0.75
Male sex	95 (59.0)	77 (55.8)	18 (78.3)	0.07
BMI (kg/m^2^)	25.7 (22.6–27.8)	25.7 (22.6–28.1)	25.3 (22.8–26.7)	0.28
Diabetes mellitus	25 (15.5)	22 (15.9)	3 (13.0)	1.00
Chronic kidney disease	70 (43.5)	61 (44.2)	9 (39.1)	0.82
CKD stage I–II	46 (28.6)	40	6	
CKD stage III	22 (13.7)	19	3	
CKD stage IV	2 (1.2)	2	0	
Ever smoker	70 (43.5)	56 (40.6)	14 (60.9)	0.11
Hospitalization in past 12 months	53 (32.9)	50 (36.2)	3 (13.0)	**0.03**
Prior GI surgery	11 (6.8)	8 (5.8)	3 (13.0)	0.19
Neoadjuvant treatment				
None	125 (77.6)	113 (81.9)	12 (52.2)	
Radiotherapy	7 (4.3)	4 (2.9)	3 (13.0)	
Chemotherapy	5 (3.1)	4 (2.9)	1 (4.3)	
Chemoradiation	24 (14.9)	17 (12.3)	7 (30.4)	
Radiotherapy-containing treatment	31 (19.3)	21 (15.2)	10 (43.5)	**0.003**
ASA class				
II	6 (3.7)	5 (3.6)	1 (4.3)	
III	110 (68.3)	97 (70.3)	13 (56.5)	0.41
IV	45 (28.0)	36 (26.1)	9 (39.1)	
PAP regimen^†^				—
Cefazolin + Metronidazole	127 (78.9)	107 (77.5)	20 (87.0)	
Other	34 (21.1)	31 (22.5)	3 (13.0)	
Surgery duration (min)	225 (180–270)	220 (180–260)	290 (180–385)	**0.008**
Days of hospitalization	8 (6–10)	7 (6–9)	10 (8–27)	**<0.001**

Data are presented as median (interquartile range) or *n* (%). *p-*values from Wilcoxon rank-sum test (continuous variables) or Fisher’s exact test (categorical variables). Bold indicates *p* < 0.05. ^†^PAP regimen presented descriptively; detailed analysis of prophylaxis compliance and outcomes reported separately. Days of hospitalisation was missing for one patient and excluded from the corresponding analysis. ASA, American Society of Anesthesiologists; BMI, body mass index; CKD, chronic kidney disease; GI, gastrointestinal; HAI, healthcare-associated infection; PAP, perioperative antibiotic prophylaxis.

**Table 2:** Patient characteristics by healthcare-associated infection (HAI) status following upper gastrointestinal surgery for cancer.

Variable	Total (*n* = 50)	No HAI (*n* = 40)	HAI (*n* = 10)	*p-*value
Age (years)	68 (58–73)	70 (59–74)	62.5 (54–72)	0.36
Male sex	33 (66.0)	26 (65.0)	7 (70.0)	1.00
BMI (kg/m^2^)	25.6 (22.7–28.0)	25.9 (23.1–28.1)	23.8 (21.5–25.3)	0.11
Diabetes mellitus	8 (16.0)	8 (20.0)	0 (0)	0.18
Chronic kidney disease	16 (32.0)	13 (32.5)	3 (30.0)	1.00
CKD stage I–II	13 (26.0)	11	2	
CKD stage III	2 (4.0)	2	0	
CKD stage IV	1 (2.0)	0	1	
Ever smoker	9 (18.0)	6 (15.0)	3 (30.0)	0.36
Hospitalization in past 12 months	39 (78.0)	31 (77.5)	8 (80.0)	1.00
Prior GI surgery	1 (2.0)	1 (2.5)	0 (0)	1.00
Neoadjuvant treatment				
None	38 (76.0)	31 (77.5)	7 (70.0)	
Chemotherapy	12 (24.0)	9 (22.5)	3 (30.0)	0.69
ASA class				
III	38 (76.0)	30 (75.0)	8 (80.0)	
IV	12 (24.0)	10 (25.0)	2 (20.0)	1.00
PAP regimen^†^				—
Cefazolin alone	29 (58.0)	22 (55.0)	7 (70.0)	
Cefazolin + Metronidazole	11 (22.0)	9 (22.5)	2 (20.0)	
Other	10 (20.0)	9 (22.5)	1 (10.0)	
Surgery duration (min)	235 (180–319)	215 (180–308)	307.5 (210–348)	0.22
Days of hospitalization	9 (8–13)	8.5 (8–11)	13.5 (11–19)	**0.002**

Data are presented as median (interquartile range) or *n* (%). *p*-values from Wilcoxon rank-sum test (continuous variables) or Fisher’s exact test (categorical variables). Bold indicates *p* < 0.05. ^†^PAP regimen presented descriptively; detailed analysis of prophylaxis compliance and outcomes reported separately. ASA, American Society of Anesthesiologists; BMI, body mass index; CKD, chronic kidney disease; GI, gastrointestinal; HAI, healthcare-associated infection; PAP, perioperative antibiotic prophylaxis.

Multivariable analysis was performed using binary logistic regression with HAI as the dependent variable. Candidate predictors were pre-specified on the basis of biological plausibility and prior literature rather than univariate significance. Three variables were included in the final model: neoadjuvant radiotherapy-containing treatment (the primary exposure of interest), surgery duration (an established risk factor for postoperative infection), and male sex (consistently reported as a risk factor for colorectal SSI and a potential confounder of the radiotherapy effect given the predominance of rectal cancer in male patients). The number of predictors was limited to respect an events-per-variable ratio of approximately 5, in line with current methodological recommendations (19). Days of hospitalization were excluded from multivariable modelling as they are partly a consequence of infection rather than an independent risk factor. Multivariable analysis was performed only for the lower GI group, as the upper GI group had insufficient events for stable modelling.

Complete-case analysis was used. Among the variables included in the descriptive tables and the multivariable model, only days of hospitalisation had a missing value (1 of 211 patients, 0.5%); no imputation was performed.

All tests were two-sided with a significance threshold of *p* < 0.05. Analyses were performed using Python 3.12 (pandas v2.1, scipy v1.11, statsmodels v0.14). PAP regimens are summarized descriptively in Tables 1, 2; a detailed evaluation of prophylaxis appropriateness, timing, and compliance in relation to infection outcomes was beyond the scope of this report and is addressed separately.

## 3 Results

### 3.1 Study population

A total of 216 patients undergoing surgery for GI cancer were consecutively included between May 2023 and July 2024. Five patients were excluded due to loss to follow-up before the 30-day assessment (all from the colorectal group). The five excluded patients were broadly comparable to the 211 analyzed patients in age (median 72 vs. 69 years), sex, diabetes (1/5), chronic kidney disease (3/5), and neoadjuvant radiotherapy (1/5); their small number precludes formal statistical comparison but argues against substantial selection bias. The analysis was performed on 211 patients: 161 in the lower GI group (colorectal cancer) and 50 in the upper GI group (45 patients with gastric cancer, 5 patients with small intestine cancer). The two groups were comparable in age (median 69 vs. 68 years) and BMI (median 25.7 vs. 25.6), and in ASA class distribution. The lower GI group had a higher proportion of ever-smokers (43% vs. 18%). Neoadjuvant treatment differed by indication: in the lower GI group, 19.3% received radiotherapy-containing regimens (radiotherapy alone or chemoradiation), while in the upper GI group neoadjuvant treatment consisted exclusively of chemotherapy (24%). Patient characteristics are presented in Tables 1, 2.

### 3.2 Incidence and types of HAI

Thirty-three of 211 patients (15.6%) developed at least one HAI within 30 days of surgery, accounting for 39 infectious episodes (26 in the lower GI surgery group and 13 in the upper GI surgery group). Twenty-seven patients (81.8%) had a single episode, while six (18.2%) had two distinct infection types. The HAI rate was 14.3% (23/161) in the lower GI group and 20.0% (10/50) in the upper GI group; this difference was not statistically significant (*p* = 0.37). SSI rates were virtually identical between groups (7.5% vs. 8.0%, *p* = 1.0).

The infection profile differed between the two groups: SSI was the predominant infection type in the lower GI surgery group, whereas device-associated infections (CVC infections) and respiratory infections (pneumonia and VAP) were relatively more common in the upper GI surgery group. Infection-attributable mortality occurred in 4 patients (1.9%), all in the lower GI surgery group.

**Figure 1:**
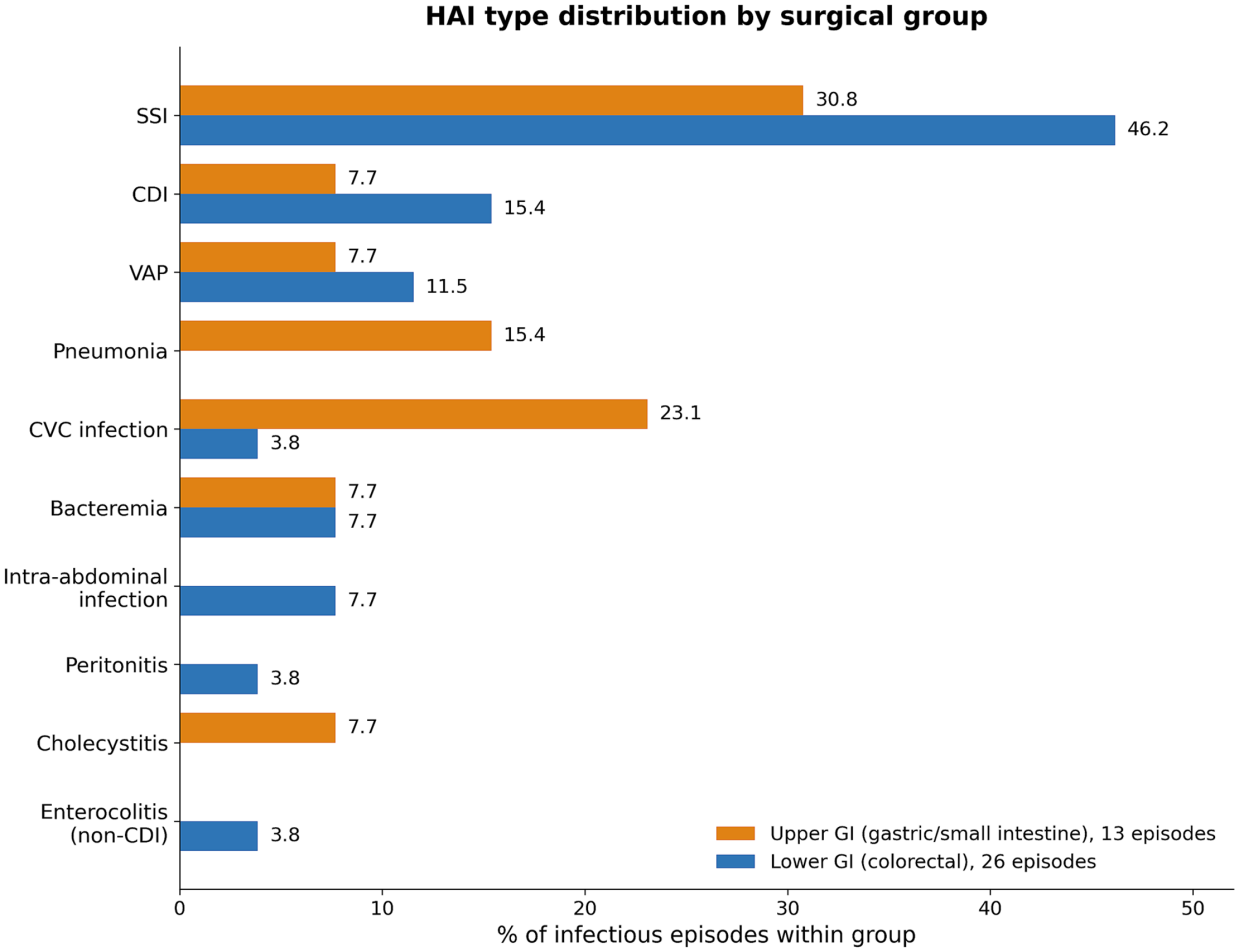
Distribution of HAI types by surgical group, shown as the percentage of infectious episodes within each group (lower GI, 26 episodes; upper GI, 13 episodes). CDI, *Clostridioides difficile* infection; CVC, central venous catheter; SSI, surgical site infection; VAP, ventilator-associated pneumonia.

The per-type distribution of HAI episodes in each group was as follows. In the lower GI surgery group (26 episodes), SSI accounted for 12 episodes (46.2%), CDI for 4 (15.4%), VAP for 3 (11.5%), intra-abdominal infection requiring reintervention without SSI for 2 (7.7%), bacteremia for 2 (7.7%), CVC infection for 1 (3.8%), peritonitis for 1 (3.8%), and non-CDI enterocolitis for 1 (3.8%). In the upper GI surgery group (13 episodes), SSI accounted for 4 episodes (30.8%), CVC infection for 3 (23.1%), pneumonia for 2 (15.4%), VAP for 1 (7.7%), CDI for 1 (7.7%), cholecystitis for 1 (7.7%), and bacteremia for 1 (7.7%) (Figure 1).

### 3.3 Risk factors for HAI: univariate analysis

In the lower GI surgery group, neoadjuvant treatment containing radiotherapy was the strongest risk factor for HAI: 43.5% of infected patients had received radiotherapy-containing regimens compared with 15.2% of uninfected patients (odds ratio OR 4.29, *p* = 0.003). Longer surgery duration was also associated with HAI (median 290 vs. 220 min, *p* = 0.008), as was longer hospitalization (median 10 vs. 7 days, *p* < 0.001). Hospitalization in the previous 12 months showed an inverse association (13.0% vs. 36.2%, OR 0.26, *p* = 0.03). Trends were observed for male sex (78.3% vs. 55.8%, *p* = 0.07) and smoking (60.9% vs. 40.6%, *p* = 0.11). Diabetes, CKD, BMI, ASA class, and prior GI surgery were not significantly associated with HAI (Table 1).

In the upper GI group, the duration of hospitalization was the only variable reaching statistical significance (median 13.5 vs. 8.5 days, *p* = 0.002). No baseline, comorbidity, or surgical variable was significantly associated with HAI in this subgroup, likely reflecting limited statistical power with 10 events in 50 patients. Non-significant trends were observed for lower BMI in infected patients (median 23.8 vs. 25.9, *p* = 0.11) and the absence of diabetes among all infected patients (0% vs. 20%, *p* = 0.18) (Table 2).

### 3.4 Risk factors for HAI: adjusted analysis

To assess the independent effect of neoadjuvant radiotherapy on HAI risk, a multivariable logistic regression model including neoadjuvant radiotherapy, surgery duration, and male sex as pre-specified predictors was performed in the lower GI group. With 23 events and 3 predictor variables, the events-per-variable ratio was 7.7. After adjustment, radiotherapy-containing treatment remained significantly associated with HAI (adjusted OR 3.25, 95% confidence interval (CI) 95% CI 1.15–9.19, *p* = 0.026), with a non-significant trend toward higher risk in male patients (adjusted OR 2.59, 95% CI 0.88–7.66, *p* = 0.084). Surgery duration did not reach significance (adjusted OR 1.18 per hour, 95% CI 0.92–1.52, *p* = 0.19). The model showed adequate discrimination (area under the receiver operating characteristic curve [AUC] 0.77) and calibration (Hosmer–Lemeshow goodness-of-fit *p* = 0.27); the radiotherapy estimate was essentially unchanged compared with a more parsimonious two-predictor model (adjusted OR 3.19), supporting its robustness to additional adjustment. An adjusted analysis was not performed for the upper GI group due to insufficient events (Table 3).

**Table 3:** Multivariable logistic regression for the association between neoadjuvant radiotherapy and healthcare-associated infection—lower gastrointestinal group (*n* = 161, 23 events).

Variable	Adjusted OR	95% CI	*p*-value
Neoadjuvant radiotherapy	3.25	1.15–9.19	**0.026**
Surgery duration (per hour)	1.18	0.92–1.52	0.19
Male sex	2.59	0.88–7.66	0.084

Multivariable logistic regression with three pre-specified predictors (neoadjuvant radiotherapy, surgery duration, and male sex). Bold indicates *p* < 0.05. Radiotherapy includes radiotherapy alone and chemoradiation. Model performance: area under the receiver operating characteristic curve 0.77; Hosmer–Lemeshow goodness-of-fit *p* = 0.27. Days of hospitalization were excluded as a potential consequence of infection rather than an independent predictor. CI, confidence interval; HAI, healthcare-associated infection; OR, odds ratio.

### 3.5 Microbiological profile

Of 33 patients with HAI, microbiological data with pathogen identification were available for 15 (45.5%)—9 (39.1%) in the lower GI surgery group and 6 (60.0%) in the upper GI surgery group—including culture results and organism identification. Rapid diagnostic tests without pathogen isolation were performed in six patients (*C. difficile* GDH and toxin assay in five patients, respiratory antigen testing in one patient). For the remaining 12 patients (36.4%), no microbiological results were recorded in the database; whether this reflects absence of sampling or negative culture results could not be determined (Supplementary Table S1).

From the 15 patients with identified pathogens, 27 bacterial and fungal isolates were recovered from wound swabs (*n* = 6 patients), catheter tips (*n* = 4), bronchial aspirates (*n* = 4), blood cultures (*n* = 3), and pleural fluid (*n* = 1). Some patients had cultures from multiple sites corresponding to distinct infectious episodes. Gram-negative organisms dominated, accounting for 25 of 27 isolates (92.6%).

Wound cultures from SSI yielded predominantly enteric *Enterobacterales*: six *Escherichia coli* isolates (one extended-spectrum *β*-lactamase (ESBL)-producing, two *Morganella morganii*, and one each of *Citrobacter freundii* and *Enterobacter cloacae*—organisms frequently recovered from colonic flora. Mixed polymicrobial growth was common, with four of six wound cultures yielding two or more species. Notably, wound cultures from one patient grew *Klebsiella pneumoniae* carrying dual carbapenemases (New Delhi metallo-*β*-lactamase (NDM) and oxacillinase-48 [OXA-48]), and from another *Pseudomonas aeruginosa* with a metallo-*β*-lactamase (MBL) alongside *Hafnia alvei*.

Bronchial aspirates from patients with VAP or pneumonia yielded non-fermenting Gram-negatives: *P. aeruginosa* XDR (2 patients), *Acinetobacter baumannii* (2 patients: 1 MDR, 1 XDR), *K. pneumoniae* XDR, and *Stenotrophomonas maltophilia*. Catheter tip cultures from CVC infections grew *P. aeruginosa* MDR with derepressed AmpC and efflux pump overexpression, *Proteus mirabilis*, and *E. cloacae*. Blood cultures identified *P. aeruginosa* MBL (1 patient), *E. coli* ESBL (1), and *Enterobacter hormaechei* (1). The single Gram-positive isolate was *Enterococcus faecalis*; the single fungal isolate, *Candida auris*, was recovered alongside *P. aeruginosa* from a bronchial aspirate in a patient who subsequently died.

**Figure 2:**
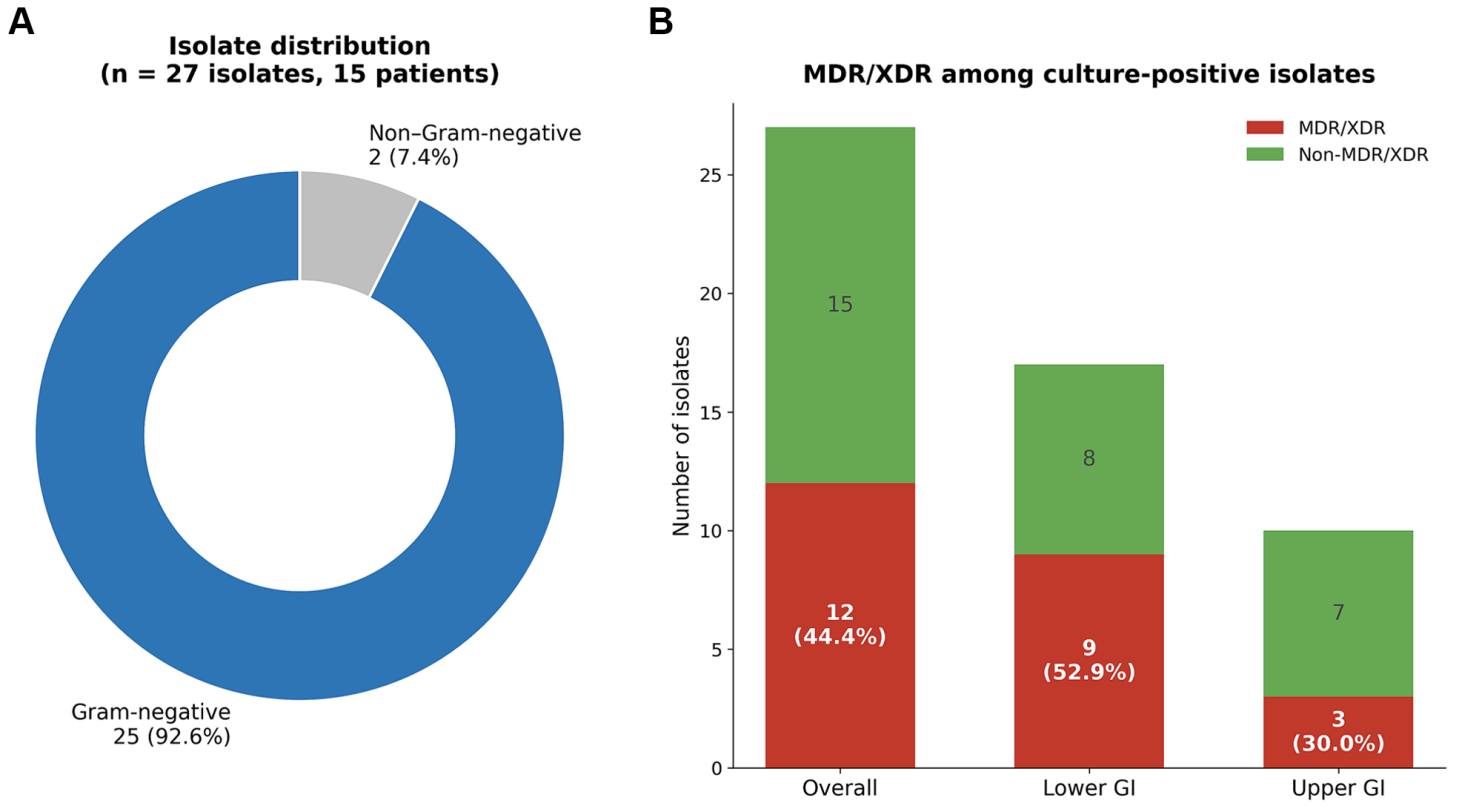
Antimicrobial resistance landscape among the 27 culture-positive isolates recovered from 15 patients. **(A)** Distribution of isolates by Gram category. **(B)** Proportion of MDR or XDR isolates overall and by surgical group, classified according to Magiorakos et al. (17).

Overall, 12 of 27 isolates (44.4%) were classified as MDR or XDR, with a higher proportion in the lower GI group (9/17, 52.9%) than in the upper GI group (3/10, 30.0%) (Figure 2). Detailed microbiological data are presented in Supplementary Tables S1, S2.

## 4 Discussion

In this prospective single-center cohort of 211 patients undergoing elective surgery for gastrointestinal cancer, the 30-day incidence of any HAI was 15.6%, accounting for 39 infectious episodes across 33 patients. SSI was the predominant infection type in the lower GI group (46.2% of episodes), whereas device-associated and respiratory infections were relatively more common in the upper GI group (CVC infections plus pneumonia together accounted for approximately 38% of episodes). Neoadjuvant radiotherapy-containing treatment emerged as an independent risk factor for HAI in the colorectal subgroup, and the microbiological profile demonstrated a substantial burden of multidrug- and XDR organisms, with carbapenemase-producing Enterobacterales and non-fermenting Gram-negatives recovered alongside isolated cases of highly resistant fungi.

The overall HAI rate observed in our cohort is consistent with rates reported in European surgical oncology populations (20), and the SSI-dominant pattern in colorectal surgery aligns with international surveillance data (21). The shift in infection type distribution between upper and lower GI surgery—with device-associated and respiratory infections relatively more common in upper GI patients—likely reflects the more invasive perioperative course of major upper GI procedures, which typically require prolonged intensive care unit (ICU) stay, more frequent mechanical ventilation, and longer central venous access (22, 23).

One of the principal findings of this study was the independent association between neoadjuvant radiotherapy-containing treatment and HAI in colorectal surgery patients (adjusted OR 3.25, 95% CI 1.15–9.19, *p* = 0.026). Should this association prove causal, several biological mechanisms could plausibly underlie it, including radiotherapy-induced mucosal injury, pelvic microvascular damage with impaired tissue healing, local immune dysfunction, and shifts in the gut microbiome that may favor colonization by resistant or opportunistic pathogens (24, 25). In brief, radiation enteropathy underlies the first two: epithelial apoptosis and mucosal-barrier breakdown facilitate bacterial translocation, while microvascular injury and tissue hypoxia impair anastomotic healing (24). Radiotherapy also causes lymphopenia and broader immunosuppression (26), which may weaken antimicrobial host defences. Most relevant to the resistant organisms seen here, radiotherapy also reduces gut microbial diversity and depletes commensal anaerobes that confer colonization resistance, permitting expansion of Enterobacterales and other opportunistic, often antibiotic-resistant taxa that may seed subsequent infection (25, 27)—a direct link between neoadjuvant radiotherapy and colonization by the resistant pathogens that predominated among our isolates. Prior studies have reported associations between neoadjuvant radiotherapy and anastomotic complications or SSI in rectal cancer (28, 29), but few have examined the broader spectrum of HAI in this population. Importantly, the radiotherapy estimate was stable when male sex—a known confounder via the predominance of rectal cancer in male patients—was added to the model, supporting the robustness of this association. Nonetheless, this estimate is based on only 23 infection events at a single center and carries a wide CI, and residual confounding cannot be excluded; the data therefore support an independent association rather than a definitive causal relationship, and confirmation in larger multicenter studies is needed before a causal role can be established. The inverse association between hospitalization in the previous 12 months and HAI on univariate analysis (OR 0.26, *p* = 0.03) is counterintuitive and warrants closer comment. Several non-exclusive explanations are plausible. First, this was one of many unadjusted univariate comparisons not corrected for multiple testing; it was not among the pre-specified predictors and did not enter the multivariable model, so a chance finding cannot be excluded. To test this directly, we re-fitted the multivariable model with prior hospitalization added to the pre-specified predictors; the association attenuated and was no longer statistically significant (adjusted OR 0.30, 95% CI 0.07–1.20, *p* = 0.09), indicating that prior hospitalization is not an independent predictor of HAI. Second, confounding by indication may operate: patients admitted in the preceding year may have undergone closer preoperative surveillance, MDRO screening, or optimization, and prior antimicrobial exposure may have transiently altered their microbiota, all of which could lower measured postoperative infection risk. Third, a healthy-survivor effect is possible, as patients fit for elective cancer surgery after a recent admission may differ systematically from those without one. We therefore interpret this association as hypothesis-generating rather than evidence of a protective effect, and one requiring prospective confirmation with detailed characterization of prior healthcare exposure.

The antimicrobial resistance landscape documented in our cohort is concerning. With 44.4% of culture-positive isolates classified as MDR or XDR, our findings reflect the well-documented high baseline of antimicrobial resistance in Romanian hospitals (10, 11). This is consistent with national surveillance: Romania reports one of the highest carbapenem-resistant *K. pneumoniae* proportions among invasive isolates in the EU (approximately 50%, third only to Greece and Bulgaria) in ECDC EARS-Net data (30); although our 44.4% aggregate MDR/XDR figure is not a like-for-like metric, it is fully in keeping with this high-resistance setting. Of particular concern were the isolated cases of carbapenem-resistant *Enterobacterales* co-producing NDM and OXA-48 carbapenemases, MBL-producing *P. aeruginosa*, and *Candida auris*, all of which represent organisms of urgent public health concern (31, 32). For Candida auris, however, this rests on a single isolate from one patient, which cannot by itself establish institutional endemicity and is better interpreted as a signal warranting active laboratory surveillance and infection-control vigilance than as evidence of local spread. The dual-carbapenemase *K. pneumoniae* carrying NDM together with OXA-48 in a single SSI underscores the local circulation of pan-resistant phenotypes and the value of complementing aggregate national or European susceptibility data with institution-specific surveillance when guiding empirical therapy for individual surgical patients in high-resistance settings. These resistance and pathogen-distribution figures should nonetheless be interpreted with caution, as they derive from the fewer than half of infected patients in whom cultures were positive and microbiological documentation was available.

The standard cefazolin-based regimen (with metronidazole for colorectal procedures) is guideline-concordant (33) and, as short-course prophylaxis, exerts limited selective pressure, but offers no activity against the ESBL- and carbapenemase-producing organisms recovered. In this high-resistance setting these most likely reflect prior colonization rather than *de novo* selection by prophylaxis, so a cefazolin-based regimen may fail to prevent infection in carriers rather than drive resistance. Risk-stratified prophylaxis for known carriers therefore merits evaluation, although a full appropriateness analysis is deferred to a dedicated report.

Beyond its magnitude, this documentation gap is best interpreted against reported surgical practice. In cohorts where specimens are systematically obtained, microbiological culture confirms a pathogen in roughly three-quarters of surgical-site infections, with only about one in 10 having no culture taken (34). Against this benchmark, the absence of any microbiological sampling in over one-third of our infected patients indicates that the gap in our cohort is driven less by culture-negativity—a well-recognized phenomenon even with optimal sampling—than by specimens not being obtained; pre-antimicrobial specimen collection is therefore the principal, actionable target for institutional quality improvement.

### 4.1 Strengths and limitations

Strengths of this study include the prospective design, the comprehensive 30-day follow-up covering the full spectrum of HAI rather than SSI alone, the standardized application of CDC/NHSN surveillance definitions, and detailed microbiological characterization including resistance mechanisms where available. Limitations include the single-center design, the limited sample size of the upper GI subgroup (*n* = 50, 10 infection events), the lack of detailed data on PAP compliance (analyzed separately in a companion stewardship paper), and the observational design which, together with the limited number of infection events, precludes causal inference for the radiotherapy association. The upper GI arm was underpowered for both modelling and between-group comparison: with only 10 HAI events among 50 patients, no multivariable analysis was feasible, and the numerically higher upper GI infection rate (20.0% vs. 14.3% in the lower GI group; *p* = 0.37) could not be reliably assessed—the study had only approximately 16% power to detect a difference of this magnitude, and roughly 680 patients per group (~1,360 total) would be required for 80% power. The comparative objective is therefore only partially achieved for the upper GI arm, and this difference should be regarded as hypothesis-generating, to be confirmed in a larger multicenter cohort. The upper GI group also combined gastric (*n* = 45) and small intestine (*n* = 5) cancers, which carry differing perioperative risk profiles; the small number of small-intestine cases precluded a separate sensitivity analysis, and this heterogeneity should be considered when interpreting the upper GI results. Most importantly, the absence of microbiological documentation in more than one-third of infected patients—with culture-positive identification in fewer than half—substantially limits the strength of our conclusions regarding pathogen distribution and antimicrobial resistance patterns, which should accordingly be regarded as indicative rather than definitive. Mortality from non-infectious causes within the 30-day window was not systematically collected, limiting the ability to compute infection-attributable fractions; one upper GI patient who died of non-infectious causes within 30 days is therefore not counted in the infection-attributable mortality rate. We also did not formally analyse temporal trends: over the 14-month period the number of events was too small to assess secular changes in HAI incidence or resistance, and the two organisms of greatest concern occurred as isolated cases—a single *C. auris* isolate and one dual-carbapenemase *K. pneumoniae*—rather than as temporal clusters, with no outbreak identified; given their recognized outbreak potential, prospective temporal surveillance is nonetheless warranted. Collectively, the single-center design, the limited sample size of the upper gastrointestinal subgroup, the incomplete microbiological documentation, and the observational nature of the study restrict the generalizability of our findings, which should be regarded as hypothesis-generating and confirmed in larger, multicenter cohorts before being extended to other centers and populations.

### 4.2 Implications and conclusions

In this Romanian tertiary-center cohort of patients undergoing elective gastrointestinal cancer surgery, the postoperative HAI burden extends substantially beyond SSI, with a differing infection profile between upper and lower GI surgery and a high antimicrobial resistance burden. Neoadjuvant radiotherapy was independently associated with HAI in colorectal cancer patients. While this association does not by itself establish causation, it supports heightened postoperative surveillance in this subgroup; whether tailored prophylaxis strategies are warranted should be evaluated in larger multicenter studies. The substantial proportion of culture-negative or unsampled HAI calls for systematic microbiological workup of postoperative infections, while the documented resistance profile—though necessarily based on the culture-positive minority of infected patients and therefore to be interpreted with caution—suggests that institution-specific surveillance data may complement broader national and European epidemiological information when developing local stewardship strategies.

More broadly, these findings should be read against the Eastern European context in which they arise. Romania carries one of the highest GI-cancer burdens (7) and among the highest carbapenem-resistance rates in the EU (30), so a tertiary surgical center here operates at the intersection of high surgical case volume and a high resistance baseline. Although single-center, the cohort is therefore likely representative of the challenges facing other Romanian and regional tertiary centers, pending multicenter confirmation. We propose a concrete research agenda: (i) multicenter prospective cohorts to confirm the HAI spectrum and the radiotherapy association; (ii) evaluation of risk-stratified prophylaxis for patients colonized with resistant organisms; and (iii) prospective temporal and molecular surveillance for outbreak-prone pathogens such as *C. auris* and carbapenemase-producing Enterobacterales. Embedding such surveillance within ongoing Eastern European health-system strengthening would help translate institution-specific data into regional infection-prevention and stewardship policy. We acknowledge, however, a practical constraint: many smaller Romanian centers lack the microbiological capacity and case volume to generate reliable institution-specific antibiograms. Where local data are insufficient, regional or networked antibiograms—pooling data across comparable centers with reference-laboratory support—offer a pragmatic alternative, and strengthening this surveillance infrastructure should itself be a policy priority.

## Data Availability

The raw data supporting the conclusions of this article will be made available by the authors, without undue reservation.

## Glossary

AmpC: AmpC *β*-lactamase
ASA: American Society of Anesthesiologists
AUC: area under the receiver operating characteristic curve
BMI: body mass index
CDC/NHSN: Centers for Disease Control and Prevention/National Healthcare Safety Network
CDI: *Clostridioides difficile* infection
CI: confidence interval
CKD: chronic kidney disease
CVC: central venous catheter
ESBL: extended-spectrum *β*-lactamase
EUCAST: European Committee on Antimicrobial Susceptibility Testing
GDH: glutamate dehydrogenase
GI: gastrointestinal
HAI: healthcare-associated infection
ICU: intensive care unit
IDSA/ATS: Infectious Diseases Society of America/American Thoracic Society
IQR: interquartile range
MBL: metallo-*β*-lactamase
MDR: multidrug-resistant
NDM: New Delhi metallo-*β*-lactamase
OR: odds ratio
OXA-48: oxacillinase-48
PAP: perioperative antibiotic prophylaxis
SSI: surgical site infection
STROBE: Strengthening the Reporting of Observational Studies in Epidemiology
VAP: ventilator-associated pneumonia
XDR: extensively drug-resistant.
