# Supplementary material for: Healthcare-associated infections after elective surgery for gastrointestinal cancer in a Romanian tertiary center: incidence, microbiological profile, and the role of neoadjuvant radiotherapy as an independent risk factor
Source: Front Public Health. 2026 Aug 5;14:1894824. doi: 10.3389/fpubh.2026.1894824 (PMC13486299; doi:10.3389/fpubh.2026.1894824)
Supplement: Supplementary file 1 [file Supplementary_file_1.DOCX]

**Supplementary Materials**

*Manuscript: Healthcare-associated infections following elective surgery for gastrointestinal cancer: a prospective single-center cohort study from Romania.*

**Supplementary Table S1.** Microbiological data availability and pathogen distribution among patients with healthcare-associated infection, stratified by surgical site.

|  | **Lower GI (n = 23)** | **Upper GI (n = 10)** | **Total (n = 33)** |
| --- | --- | --- | --- |
| ***Microbiological data availability*** | | | |
| Pathogen identified (culture) | 10 | 5 | 15 (45.5%) |
| Diagnostic test only | 4 | 2 | 6 (18.2%) |
| No microbiological data recorded | 9 | 3 | 12 (36.4%) |
| ***Isolates from culture-positive patients (n = 15 patients, 27 isolates)*** | | | |
| **Gram-negative** | 14 | 10 | **24 (88.9%)** |
| *Escherichia coli* | 4 | 1 | 5 |
| *Escherichia coli* ESBL | 1 | 0 | 1 |
| *Pseudomonas aeruginosa* | 3 | 3 | 6 |
| MBL | 1 | 1 | 2 |
| XDR | 0 | 2 | 2 |
| MDR (AmpC + efflux) | 1 | 0 | 1 |
| MDR | 1 | 0 | 1 |
| *Enterobacter cloacae* | 1 | 1 | 2 |
| *Morganella morganii* | 2 | 0 | 2 |
| *Klebsiella pneumoniae* | 2 | 0 | 2 |
| XDR | 1 | 0 | 1 |
| NDM + OXA-48 | 1 | 0 | 1 |
| *Acinetobacter baumannii* | 2 | 0 | 2 |
| MDR | 1 | 0 | 1 |
| XDR | 1 | 0 | 1 |
| *Citrobacter freundii* | 1 | 0 | 1 |
| *Proteus mirabilis* | 0 | 1 | 1 |
| *Hafnia alvei* | 0 | 1 | 1 |
| *Stenotrophomonas maltophilia* | 0 | 1 | 1 |
| *Enterobacter homaechei* | 0 | 1 | 1 |
| **Gram-positive** | 0 | 1 | **1 (3.7%)** |
| *Enterococcus faecalis* | 0 | 1 | 1 |
| **Fungi** | 1 | 0 | **1 (3.7%)** |
| *Candida auris* | 1 | 0 | 1 |
| **Total isolates** | **17** | **10** | **27** |
| **MDR/XDR isolates** | 9 (52.9%) | 3 (30.0%) | **12 (44.4%)** |
| ***Infection-attributable 30-day mortality among HAI patients*** | | | |
| Infection-attributable death | 4 (17.4%) | 0 (0%) | 4 (12.1%) |

ESBL = extended-spectrum β-lactamase; MBL = metallo-β-lactamase; MDR = multidrug-resistant; XDR = extensively drug-resistant; NDM = New Delhi metallo-β-lactamase; OXA-48 = oxacillinase-48; AmpC = chromosomal cephalosporinase. Diagnostic tests include *Clostridioides difficile* GDH/toxin assay (5 patients) and respiratory antigen testing (1 patient). Resistance mechanisms listed where identified from laboratory reports; intrinsic resistance assessed per EUCAST Expert Rules. Mortality denominators: 23 lower GI HAI patients, 10 upper GI HAI patients, 33 total HAI patients. One additional upper GI death (patient 10-15) was attributed to non-infectious causes and is not included in this rate.

**Supplementary Table S2.** Microbiological detail of all patients with healthcare-associated infection (n = 33).

| **ID** | **Group** | **Ep.** | **Infection type** | **Sample** | **Organisms** | **Micro** | **Outcome** |
| --- | --- | --- | --- | --- | --- | --- | --- |
| ***Lower gastrointestinal (colorectal) — 23 patients, 26 episodes*** | | | | | | | |
| **12-2** | LGI | 1 | Intra-abd. inf. (reintervention) | — | — | No data | **Died (inf.-attributable)** |
| **12-9** | LGI | 1 | Enterocolitis (non-CDI) | GDH test | GDH/toxin negative | Test neg | Survived |
| **12-10** | LGI | 1 | SSI (persistent) | — | — | No data | Survived |
| **12-18** | LGI | 1 | SSI | — | — | No data | Survived |
| **12-22** | LGI | 1 | SSI | Wound swab | *Morganella morganii* + *E. coli* | Positive | Survived |
| **12-29** | LGI | 1 | SSI | — | — | No data | Survived |
| **12-45** | LGI | 1 | VAP | Bronchial asp. | *P. aeruginosa* MDR + *Candida auris* | Positive | **Died (inf.-attributable)** |
| **12-52** | LGI | 1 | Intra-abd. inf. (reintervention) | — | — | No data | **Died (inf.-attributable)** |
| **12-59** | LGI | 1 | SSI | — | — | No data | **Died (inf.-attributable)** |
| **12-64** | LGI | 2 | CDI + Bacteremia | GDH+toxin; Blood cx | *C. difficile* (GDH+toxin +); *E. coli* ESBL | Test +; Pos. | Survived |
| **12-65** | LGI | 1 | SSI | — | — | No data | Survived |
| **12-70** | LGI | 2 | CVC inf. + VAP | Catheter tip; Bronchial asp. | *P. aeruginosa* MDR AmpC+efflux (CVC); *A. baumannii* MDR (VAP) | Positive | Survived |
| **12-81** | LGI | 1 | CDI | GDH+toxin | *C. difficile* (GDH+toxin +) | Test + | Survived |
| **12-103** | LGI | 1 | Peritonitis | Pleural fluid | *E. coli* | Positive | Survived |
| **12-104** | LGI | 1 | SSI | — | — | No data | Survived |
| **12-107** | LGI | 1 | CDI | GDH+toxin | *C. difficile* (GDH+toxin +) | Test + | Survived |
| **12-108** | LGI | 1 | SSI | Wound swab | *E. coli* + *C. freundii* | Positive | Survived |
| **12-119** | LGI | 1 | SSI | Wound swab | *Morganella* + *E. coli* + *E. cloacae* | Positive | Survived |
| **12-129** | LGI | 1 | CDI | GDH+toxin | *C. difficile* (GDH+toxin +) | Test + | Survived |
| **12-131** | LGI | 1 | Bacteremia | Blood culture | *P. aeruginosa* MBL | Positive | Survived |
| **12-141** | LGI | 1 | SSI (persistent) | — | — | No data | Survived |
| **12-149** | LGI | 1 | SSI | — | — | No data | Survived |
| **12-151** | LGI | 2 | VAP + SSI | Bronchial asp.; Wound swab | *K. pneumoniae* XDR + *A. baumannii* XDR (VAP); *K. pneumoniae* NDM+OXA-48 (SSI) | Positive | Survived |
| ***Upper gastrointestinal (gastric + small intestine) — 10 patients, 13 episodes*** | | | | | | | |
| **10-1** | UGI | 1 | SSI | Wound swab | *E. faecalis* + *E. coli* | Positive | Survived |
| **10-3** | UGI | 1 | CDI | GDH+toxin | *C. difficile* (GDH+toxin +) | Test + | Survived |
| **10-9** | UGI | 1 | Pneumonia | Antigen test | COVID Ag neg; Flu Ag neg | Test neg | Survived |
| **10-12** | UGI | 1 | CVC inf. | Catheter tip | *Proteus mirabilis* | Positive | Survived |
| **10-15** | UGI | 2 | Cholecystitis + VAP | — (cholecyst.); Bronchial asp. (VAP) | — (cholecyst.); *P. aeruginosa* XDR + *S. maltophilia* (VAP) | No data; Pos. | **Died (non-infectious)** |
| **10-18** | UGI | 2 | Pneumonia + CVC inf. | Catheter tip | *P. aeruginosa* XDR (both episodes) | Positive | Survived |
| **10-22** | UGI | 1 | SSI (persistent) | — | — | No data | Survived |
| **10-28** | UGI | 1 | SSI | — | — | No data | Survived |
| **10-30** | UGI | 2 | SSI + CVC inf. | Wound swab; Catheter tip | *P. aeruginosa* MBL + *Hafnia alvei* (SSI); *E. cloacae* (CVC) | Positive | Survived |
| **10-38** | UGI | 1 | Bacteremia | Blood culture | *Enterobacter homaechei* | Positive | Survived |

LGI = lower gastrointestinal; UGI = upper gastrointestinal; Ep. = number of distinct infectious episodes per patient; CDI = *Clostridioides difficile* infection; CVC = central venous catheter; VAP = ventilator-associated pneumonia; SSI = surgical site infection; Intra-abd. inf. (reintervention) = intra-abdominal infection requiring surgical re-exploration without surgical site infection meeting CDC/NHSN criteria; MBL = metallo-β-lactamase; MDR = multidrug-resistant; XDR = extensively drug-resistant; ESBL = extended-spectrum β-lactamase; NDM = New Delhi metallo-β-lactamase; OXA-48 = oxacillinase-48; AmpC = chromosomal cephalosporinase. **Micro:** Positive (Pos.) = pathogen identified by culture; Test + = positive rapid diagnostic test (GDH/toxin); Test neg = negative rapid diagnostic test; No data = no microbiological results recorded. Persistent = same infection type at multiple follow-up visits, counted as a single episode. **Outcome:** infection-attributable death determined by clinical assessment; one patient (10-15) died of non-infectious causes within 30 days and is not counted in the infection-attributable mortality rate.
